# Semicircular canals in *Anolis* lizards: ecomorphological convergence and ecomorph affinities of fossil species

**DOI:** 10.1098/rsos.170058

**Published:** 2017-10-11

**Authors:** Blake V. Dickson, Emma Sherratt, Jonathan B. Losos, Stephanie E. Pierce

**Affiliations:** 1 Museum of Comparative Zoology and Department of Organismic and Evolutionary Biology, Harvard University, Cambridge, MA, USA; 2 School of Biological Sciences, The University of Adelaide, Adelaide, South Australia, Australia

**Keywords:** inner ear, bony labyrinth, geometric morphometrics, three-dimensional, anoles, ecomorphology

## Abstract

*Anoli*s lizards are a model system for the study of adaptive radiation and convergent evolution. Greater Antillean anoles have repeatedly evolved six similar forms or ecomorphs: crown-giant, grass-bush, twig, trunk, trunk-crown and trunk-ground. Members of each ecomorph category possess a specific set of morphological, ecological and behavioural characteristics which have been acquired convergently. Here we test whether the semicircular canal system—the organ of balance during movement—is also convergent among ecomorphs, reflecting the shared sensory requirements of their ecological niches. As semicircular canal shape has been shown to reflect different locomotor strategies, we hypothesized that each *Anolis* ecomorph would have a unique canal morphology. Using three-dimensional semilandmarks and geometric morphometrics, semicircular canal shape was characterized in 41 *Anolis* species from the Greater Antilles and the relationship between canal shape and ecomorph grouping, phylogenetic history, size, head dimensions, and perch characteristics was assessed. Further, canal morphology of modern species was used to predict the ecomorph affinity of five fossil anoles from the Miocene of the Dominican Republic. Of the covariates tested, our study recovered ecomorph as the single-most important covariate of canal morphology in modern taxa; although phylogenetic history, size, and head dimensions also showed a small, yet significant correlation with shape. Surprisingly, perch characteristics were not found to be significant covariates of canal shape, even though they are important habitat variables. Using posterior probabilities, we found that the fossil anoles have different semicircular canals shapes to modern ecomorph groupings implying extinct anoles may have been interacting with their Miocene environment in different ways to modern *Anolis* species.

## 1. Introduction

The semicircular canals are a functional component of the vestibular system of the inner ear that enable vertebrate animals to coordinate fast and complex movements in three-dimensions (3D). As such, it stands to reason that more agile and mobile animals, such as fast moving arboreal species, would benefit from an enhanced sense of balance, granted through adaptation of canal morphology. Recent theoretical [1,2], physiological [3,4] and comparative [5–9] studies have shown that, in mammals, a relationship exists between canal morphology and vestibular sensitivity [1–4], locomotor activity [8,9], agility [7] and speed [6,10]. Although these studies use different metrics for canal morphology, such as canal size [7,11], torsion [5] and orthogonality [2,6], all agree there is a strong signal between semicircular canal morphology and locomotor ability. Thus, our current understanding of the vestibular system in living mammals has allowed us to investigate and interpret the behaviour and ecology of extinct species [12–22].

The vestibular system has, however, been little explored outside the Mammalia. While the semicircular canals are physiologically and anatomically homologous in all vertebrates, we cannot assume that the relationship between form and function in mammals will hold true for other taxa, especially given considerable morphological differences between mammals and other amniote groups. Extrapolation from mammals is particularly problematic for studies wishing to reconstruct the ecology of non-mammalian fossil species [21,23–26]. Recent studies looking at the morphology of the semicircular canals with respect to ecology in amphibians [27], squamates [28,29] and birds [30,31] have begun to expand our knowledge of the vestibular system beyond mammals, though much work is still needed to fully understand the system from a greater evolutionary and ecological spectrum.

*Anolis* lizards (Dactyloidae) represent a unique opportunity for furthering such research. Greater Antillean anoles originated ca. 65 Ma, diversifying throughout the Caribbean and neotropical mainland [32]. Within *Anolis*, six ecomorph types have evolved independently on each of the four Greater Antilles islands (with several exceptions), with each ecomorph encompassing a specific suite of anatomical (e.g. short versus long limbs), ecological (e.g. tree trunk versus branches), and behavioural (e.g. locomotion, territoriality) characteristics (reviewed in [33]): trunk-ground (TG) ecomorphs inhabit lower tree trunks, using infrequent, rapid descents to the ground where they capture prey; trunk-crown (TC) ecomorphs occupy the upper reaches of the tree, navigating the complex 3D canopy with a high rate of movement; trunk (Tr) ecomorphs occupy the trunk area between TG and TC ecomorphs with some overlaps, and are fairly active locomotors; crown-giant (CG) ecomorphs occupy similar habitats to TC ecomorphs, yet are substantially larger, and move more slowly; twig (Tw) ecomorphs occupy the narrowest branches and twigs of the canopy moving regularly, yet slowly; and grass-bush (GB) ecomorphs are also found on narrow vegetation, but close to the ground on grasses, bushes and small trees, navigating their complex 3D environment slowly. The fossil record of anoles is extremely limited, though rich Dominican Miocene amber deposits preserve at least several ecomorph types ca. 23 Ma [34].

The recurrent and consistent adaptive radiations found in *Anolis*, and close phylogenetic relatedness, make it an excellent starting point for further understanding the morpho-functional relationship of the semicircular canals in squamates and beyond Mammalia in general. Here, we use 3D geometric morphometrics to quantify and investigate whether *Anolis* semicircular canal morphology is convergent among ecomorph groups. Specifically, we aim to test the hypothesis that anole species adapted to similar ecomorph niches have converged on similar semicircular canal morphologies, reflecting the sensory requirements of their shared ecological and behavioural habits. We also test the influence of phylogenetic relatedness, size, and head proportions on patterns of canal morphology, factors that may have an effect on vestibular system form [7,9,28,35–39], as well as perch height and diameter—the two most frequently reported habitat variables [33]. Further, we reconstruct the vestibular system in five 15–20 Ma fossil anole specimens preserved in Miocene amber [34]. We use our extant dataset to predict the ecomorph affinities and palaeoecology of these extinct *Anolis* lizards and compare these to predictions based on external morphological traits [34].

## 2. Material and methods

### 2.1. Specimens and sample size

**Figure 1.**
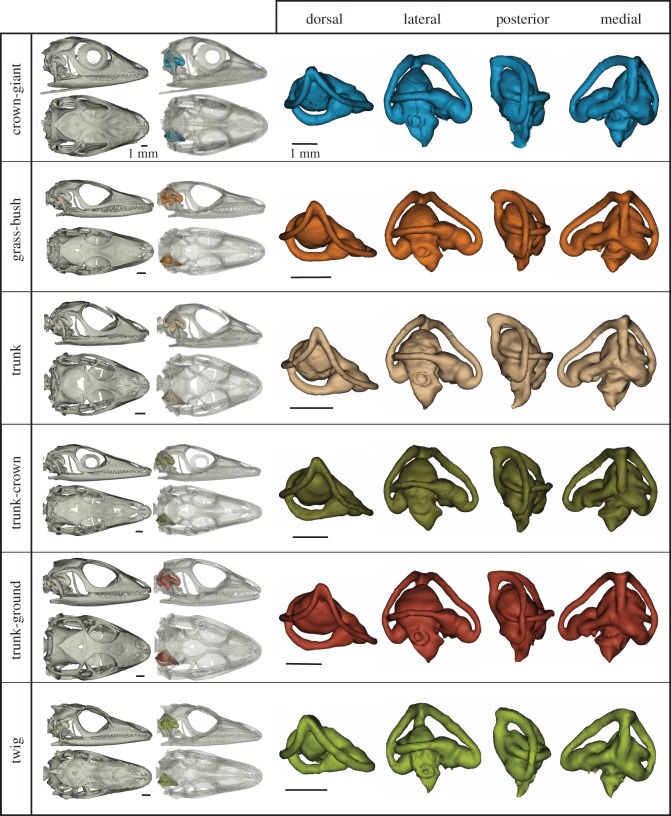
3D rendering of the vestibular system from the six modern *Anolis* ecomorphs. Each ecomorph is represented by the specimen closest to the group shape mean: crown-giant—*A. riccordii* MCZ R83982; grass-bush—*A. hendersoni* MCZ R65643; trunk-crown—*A. longiceps* MCZ R16194; trunk-ground—*A. marcanoi* MCZ R104402; trunk—*A. brevirostris* MCZ R155833; twig—*A. insolitus* MCZ R128310. MCZ, Museum of Comparative Zoology, Harvard. See the electronic supplementary material, table S1 for full specimen list. Scale bar = 1 mm.

**Figure 2.**
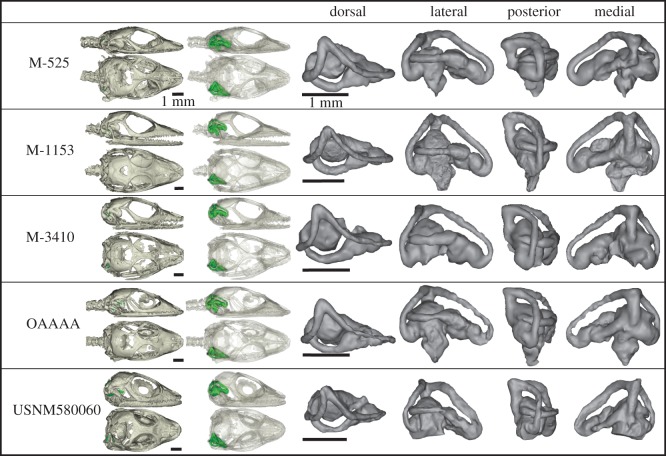
3D rendering of the five Dominican Republic anole fossil specimens, preserved in Miocene amber. The vestibular system of each specimen is shown within the skull, and in four anatomical views. See the electronic supplementary material, table S1 for full specimen identitys. Scale bar = 1 mm.

The sample consists of 131 individuals representing 41 species of anoles originating from the four islands of the Greater Antilles: Hispaniola, Cuba, Jamaica and Puerto Rico. Species from Hispaniola are represented by multiple specimens including juvenile individuals (see further below). All six ecomorphs are represented by multiple species: CG (5), GB (6), Tr (3), TC (9) TG (4) and Tw (5) (figure 1), with the addition of eight ‘unique’ species that are endemic to each island but do not form a coherent group, nor conform ecomorphologically to any of the specified ecomorphs [33]. Five fossil anoles of Miocene age (figure 2) were also included from the amber deposits of the Dominican Republic [34,40], details of which can be found in Sherratt *et al.* [34]. All modern specimens were sourced from the Herpetology collection at the Museum of Comparative Zoology (MCZ), Harvard University. All species and specimen numbers can be found in the electronic supplementary material, table S1.

### 2.2. Data acquisition and landmarks

Various methods for measuring the complex structure of the three semicircular canals have been used to date. Traditional morphometric approaches, in the form of linear and angular measures of size and orthogonality, have been used extensively in the past [2,6,7,11] with the benefit of being easily comparable across studies. However, these measurements struggle to completely capture the full shape variation in the canals owing to their complex curvature. Geometric morphometrics (GM; [41,42]) has been used increasingly to overcome this shortcoming, with landmark [9,16,28,35] and semilandmark [19,43,44] approaches, particularly the latter, capable of capturing far more morphological variation than standard morphometrics. GM methods do, however, vary between studies and are thus less easily compared across studies and broader taxonomic groups. For morphometric approaches, digital thresholding and segmenting of micro computed tomography (µCT) data can introduce significant variation in canal lumen thickness [45], making measuring and digitizing the canal surface error prone. Instead, using a centreline through the lumen overcomes this potential error as it is not affected by threshold values [43,45]. This is the approach that we took.

**Figure 3.**
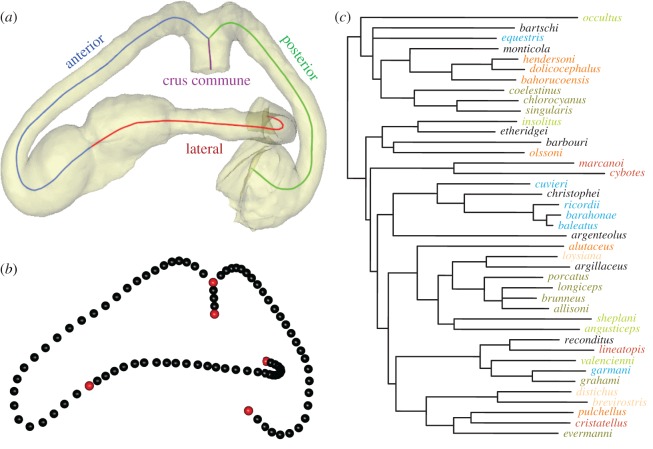
Segmented semicircular canal demonstrating placement of (*a*) centrelines and (*b*) semilandmarks; and (*c*) a time calibrated phylogeny of study taxa coloured by ecomorph. Red landmarks are fixed, while black landmarks are sliding. Species colour coding: blue = crown-giant; orange = grass-bush; pink = trunk; army green = trunk-crown; red = trunk-ground; bright green = twig; black = unique.

Specimens were µCT scanned using a variety of imaging systems and settings (electronic supplementary material, table S1) and the semicircular canals manually segmented and 3D rendered using Materialise Mimics® software. 3D landmarks were derived from the centreline of the semicircular canals, calculated using the ‘medcad’ module in Mimics®, and then manually adjusted to optimize the position of the centreline through the lumen. This centreline was then split into four segments—the three canals (anterior, posterior and lateral) and the crus commune (figure 3*a*)—and exported as 3D coordinates describing a curve. The centreline curves were then resampled using the Resample executable [46] so that the three canals were each described by 28 equally-spaced semilandmarks, and the crus commune by three semilandmarks. These semilandmarks were anchored by five landmarks positioned at the junction of the anterior and lateral canals, the posterior and lateral canals with the vestibule, and the junction of the crus commune, resulting in 92 landmarks in total (figure 3*b*).

### 2.3. Data analysis

Landmark coordinate data were aligned by Procrustes superimposition, allowing semilandmarks to slide along their tangent directions in order to minimize bending energy [47,48] using the R statistical environment v. 3.2.3 [49] and the package *geomorph* v. 2.1.4 [50]. The resulting Procrustes residuals were used as shape variables in the subsequent analyses. Alignment was done on both the full shape variable dataset (*n* = 131) and a phylogenetic subset (*n* = 41). Since the full dataset represented ontogenetic series of each species, the phylogenetic subset was represented by the largest adult male from each species paired with the phylogeny of Gamble *et al*. [51] (figure 3*c*). As a previous study found significant sexual dimorphism in some *Anolis* species [52], comparing all males avoids a potential source of bias. Partitioning the data was necessary to investigate the role phylogeny might play in determining canal shape as no current methods are available that would account for ontogenetic variation in phylogenetic comparative analyses.

Principal component analysis (PCA) was performed on the phylogenetic subset dataset to visualize semicircular canal shape variation among all species. Eigendecomposition of this PCA was performed using only modern taxa (including unique species); fossil specimens were later projected into this morphospace by matrix multiplication with the PCA eigenvectors. MANOVA was conducted to assess which PC axes significantly separated ecomorph groups. The phylogeny [51] was projected into the phylogenetic subset PC space to visualize the estimated evolutionary trajectory of canal shape change, using the *geomorph* function ‘plotGMPhyloMorphoSpace’. Phylogenetic signal of canal morphology was calculated using the K statistic [53,54] with *geomorph*'s ‘physignal’ function and tested for significance using 10 000 permutations. To visualize a morphospace independent of the effects of phylogeny and allometry, we plotted the residuals of a phylogenetic regression with log-transformed semicircular canal centroid size, performed on PC scores using the ‘phyl.resid’ function of *phytools* v. 0.5-10 [55,56]. Centroid size is a measure of size calculated as the square root of the sum of squared distances of a set of landmarks from their centroid [42]. To visualize shape changes throughout the PCA morphospace, partial warps were used to generate maximum and minimum shape warps along each principal component (PC) axis by back-transformation through the eigenvectors.

To determine whether ecomorphs occupy different regions of morphospace (and thus have significantly different canal morphologies), and to test the effect of size and head proportions on canal shape, analysis of covariance (ANCOVA) and phylogenetic generalized least squares (PGLS) were performed on the phylogenetic dataset using the ‘procD.lm’ and ‘procD.pgls’ functions respectively [57] of *geomorph*, with pairwise comparisons tested using ‘advanced.procD.lm’ [50]*.* These functions perform statistical assessment of the terms in the model using Procrustes distances among specimens, rather than explained covariance matrices among variables, and are thus suitable for multivariate datasets [57,58]. Three log-transformed size metrics were used: semicircular canal centroid size, skull length (measured between the premaxilla and the dorsal margin of the foramen magnum) and skull width (measured between the paraoccipital processes). The relationship between the size metrics was also explored using linear regressions. Head proportions were determined by taking the ratio of skull length : width. In addition, we investigated the relationship between canal shape and habitat use, represented by perch height and diameter ([50] and J. B. Loses 1988–2005, unpublished)—the two most frequently reported habitat variables—by ANCOVA and PGLS.

Finally, a canonical variate analysis (CVA) with cross-validations was run using the ‘CVA’ function of the package *Morpho* v. 2.3.0 [59] to explore the morphological shape variables that maximize between-ecomorph-group variance relative to within-group variance, and to predict the potential ecology of the fossil anoles. Prior to running the CVA, a PCA was performed on the full extant dataset (excluding unique species) and the first 40 PC axes representing 99% of the variation were extracted; this reduction in dimensionality was done to ensure that the number of shape variables (*n* = 40) was less than the number of individual specimens (*n* = 99) [60] and to remove minor components of shape variance that might be attributable to error. In addition, the full specimen dataset was used to take into consideration both specific and ontogenetic variation to compare against the fossil specimens and to increase the power of the test by incorporating a larger sample size. Within the CVA morphospace, 95% confidence intervals (CI) were generated around each of the modern ecomorph groups. The unique and fossil specimens were then projected into this morphospace using the canonical variates. As CVA is not a rigid ordination and the resulting morphospace may deviate from a Euclidean space, we use Mahalanobis distances in subsequent analyses to correct for any distortions in shape-space [60]. The posterior (typicality) probability of ecomorph-group membership for fossil and unique specimens was assessed by calculating the Mahalanobis distances of each specimen to the mean of each ecomorph. This distance was then compared to within-ecomorph-group distances which had been resampled 10 000 times [61–65]. If the distance between a specimen and group mean was greater than 95% (*p* < 0.05) of within-group distances, we reject the null-hypothesis that it belongs to that ecomorph group [61,65–68]. Further, log-likelihood estimations were also calculated to allow comparison with previous work [34]. To visualize shape changes throughout the CVA morphospace, partial warps were used to generate maximum and minimum shape warps along each canonical variate (CV) axis [60].

## 3. Results

### 3.1. Patterns of shape variation

**Figure 4.**
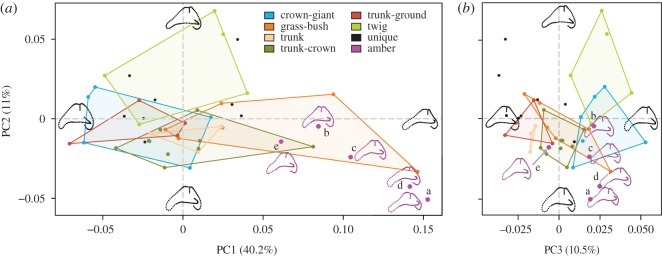
Principal component (PC) analysis of semicircular canal shape showing (*a*) PC1 versus PC2 and (*b*) PC2 versus PC3. The first three PCs represent 61.7% of variation in canal shape. Points are specimens, coloured by ecomorph and bounded by convex hulls. Unique specimens are shown in black, and fossil specimens in magenta. Partial warps representing the maxima and minima of each PC are shown on each axis, and partial warps of the fossils are shown in magenta. Anterior canal is to the left. Amber fossils: a = M-525, b = M-1153, c = M-3410, d = OAAAA, e = USNM580060.

PCA (figure 4*a*,*b*) shows that PC1 (40.2% of variation) largely represents changes in anterior and lateral canal morphology. Moving from PC1 positive to PC1 negative, there is a trend for the canals to become more rounded and anterodorsally shortened. PC2 (11.0% of variation) represents moderate changes in all three canals, with a PC2 positive to PC2 negative shift showing rounding of the anterior-most section of the anterior canal, more torsion (out-of-plane curvature) of the lateral canal, and less torsion of the posterior canal. PC3 (10.5% of variation) represents changes in the anterior and posterior canals, with a transition from PC3 positive to PC3 negative showing increased curvature and deepening of the posterior canal and reduction of the lateral aspect of the anterior canal. MANOVA results show that ecomorphs at not distinct on PC1, but they do significantly separate over subsequent PCs (electronic supplementary material, table S2).

Visually, there is significant overlap between ecomorph groupings along PC1 and PC2, with GB anoles occupying most of PC1. PC3 separates the three ‘trunk’ ecomorphs from the Tw and CG ecomorphs. When the shape data were corrected for size and phylogeny, the PC morphospace is minimally altered. The unique species are widely distributed across morphospace, overlapping with most ecomorph grouping (PC2 versus PC1) and falling outside the variation enclosed by the ecomorphs (PC2 versus PC3). All fossil specimens fall along the positive end of PC1, in the GB area of morphospace, which represents flattening and anterodorsal elongation of the anterior canal. Furthermore, three fossil specimens (M-1153, M-3410, USNM580060) overlap with multiple ecomorph groupings along PC3. Fossil specimens M-525 and OAAAA appear to fall beyond the morphologies of all living taxa sampled.

### 3.2. Predictors of shape

**Figure 5.**
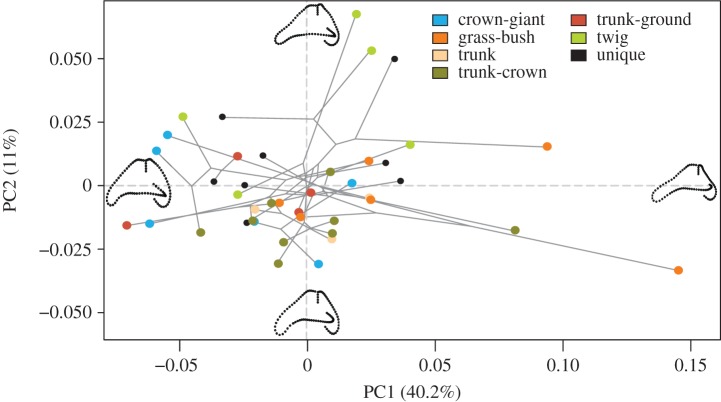
Time calibrated *Anolis* phylogeny projected into the morphospace of PC1 versus PC2, representing 51.2% of the total variation. Points are coloured by ecomorph, with unique species in black. Partial warps representing the maxima and minima of each PC are shown in black on each axis.

Phylogeny was found to have only a weak influence on semicircular canal morphology (*K* = 0.58), though permutation found this influence to be greater than expected from random (*p* = 0.0034). Mapping of the phylogeny onto morphospace (figure 5) shows extensive overlapping of branches through morphospace, indicating convergence towards similar semicircular canal morphologies.

ANCOVA (table 1) found that ecomorph is a moderate and significant predictor (*R*^2^ = 0.36, *p* < 0.001) of canal shape. Multivariate pairwise *post hoc* tests found all ecomorphs to be significantly different (*p* < 0.05) from one another except Tr and TG (*p* = 0.300, electronic supplementary material, table S3). A weak but significant relationship also exists between canal centroid size and canal shape (*R*^2^ = 0.11, *p* = 0.001) and their interaction (*R*^2^ = 0.11, *p* < 0.001), as well as head proportions and canal shape (*R*^2^ = 0.03, *p* = 0.041) and their interaction (*R*^2^ = 0.23, *p* < 0.001) (table 1). Further, PGLS (table 2) found ecomorph to be a significant predictor of canal shape, though the effect was less strong (*R*^2^ = 0.20, *p* < 0.001). This reduction in the correlation coefficient indicates an interaction between phylogeny and ecomorph and that ecomorph groupings are not entirely independent of phylogeny. PGLS (table 2) also reveals a relationship between canal centroid size and shape (*R*^2^ = 0.19, *p* < 0.001) and an interaction between ecomorph and centroid size (*R*^2^ = 0.19, *p* < 0.001). Head proportions remains weak, yet significant (*R*^2^ = 0.08, *p* = 0.006), though PGLS reveals a much stronger interaction between head proportions and ecomorph (*R*^2^ = 0.3, *p* = 0.002). There was no significant relationship between either perch height or diameter and canal shape, with and without phylogenetic correction (tables 1 and 2).

**Table 1.** Analysis of covariance (ANCOVA) of semicircular canal shape against ecomorph, semicircular canal centroid size, head proportion and perch characteristics, with statistical significance assessed through 10 000 permutations. (Significant (*p* < 0.05) results are indicated in bold.)

	d.f.	SS	MS	*R*^2^	*F*	*Z*	*p*-value
ANCOVA (ecomorph and centroid size)							
ecomorph	5	0.042349	0.0084698	0.36273	3.7724	4.5749	**0**.**0002**
centroid size	1	0.013328	0.0133283	0.11416	5.9364	4.353	**0**.**0001**
ecomorph : centroid size	5	0.013925	0.002785	0.11927	1.2404	3.16	**0**.**0007**
residuals	21	0.047149	0.0022452				
total	32	0.116751					
ANCOVA (ecomorph and head proportions)							
ecomorph	5	0.042349	0.0084698	0.36273	3.9739	4.7372	**0**.**0001**
head proportion	1	0.00331	0.0033099	0.02835	1.553	1.907	**0**.**0412**
ecomorph : head proportion	5	0.026334	0.0052668	0.22556	2.4711	4.5055	**0**.**0001**
residuals	21	0.044758	0.0021313				
total	32	0.116751					
ANCOVA (perch characteristics)							
height	1	1446	1446	0.000701	0.0195	0.0143	0.9946
diameter	1	39212	39212	0.019007	0.5283	0.42849	0.60954
height : diameter	1	18447	18447	0.008942	0.2486	0.16515	0.75882
residuals	27	2003863	74217				
total	30	2062967

**Table 2.** Phylogenetic generalized least squares of semicircular canal shape against ecomorph, semicircular canal centroid size, head proportion and perch characteristics, with statistical significance assessed through 10 000 permutations. (Significant (*p* < 0.05) results are indicated in bold.)

	d.f.	SS	MS	*R*^2^	*F*	*Z*	*p-*value
PGLS (ecomorph and centroid size)							
ecomorph	5	0.11454	0.022909	0.19789	1.9571	3.3133	**0.0002**
centroid size	1	0.10885	0.108846	0.18804	9.2985	4.3487	**0.0001**
ecomorph : centroid size	5	0.10751	0.021501	0.18572	1.8368	3.9763	**0.0002**
residuals	21	0.24582	0.011706				
total	32	0.57884					
PGLS (ecomorph and head proportions)							
ecomorph	5	0.11454	0.022909	0.197885	1.9964	3.2648	**0.0006**
ratio	1	0.04476	0.044762	0.077331	3.9009	2.7617	**0.0064**
ecomorph : ratio	5	0.17644	0.035287	0.304808	3.0751	3.2949	**0.0021**
residuals	21	0.24097	0.011475				
total	32	0.57884					
PGLS (perch characteristics)							
height	1	0.03675	0.036751	0.086641	3.6934	0.81864	0.45145
diameter	2	0.05866	0.029329	0.138286	2.9475	0.4274	0.91281
height : diameter	3	0.08996	0.029986	0.212076	3.0135	0.37058	0.94441
residuals	24	0.23881	0.00995				
total	30	0.42417					

### 3.3. Ecomorph differences

**Figure 6.**
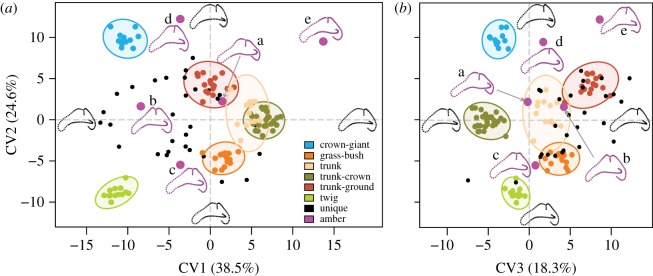
Canonical variates (CV) analysis showing (*a*) CV1 versus CV2, and (*b*) CV2 versus CV3, representing 84.8% of the total variation. Ninety-five per cent confidence ellipses of each ecomorph are plotted, and points are coloured by ecomorph, with unique species plotted in black and fossil specimens in magenta. Partial warps representing the maxima and minima of each CV are shown in black along each axis. Anterior canal is to the left. Amber fossils: a = M-525, b = M-1153, c = M-3410, d = OAAAA, e = USNM580060.

The CVA biplots (figure 6) and posterior probabilities (table 3) show ecomorphs form significantly different groups within the morphospace (*p* < 0.0001), with a cross-validation accuracy of 87.9% (electronic supplementary material, table S4). On the extreme negative end of canonical function 1 (CV1, figure 6*a*) are the Tw and CG ecomorphs. This region of morphospace is characterized by greater out-of-plane curvature of the anterior canal. The two groups, however, occupy opposite extremes of CV2 (figure 6*a*,*b*). CGs occupy the extreme positive end of CV2 and, when compared with Tw species, display greater dorsoventral curvature and reduced torsion of the posterior canal, and lengthening of the crus-commune. GB and the three ‘trunk’ ecomorphs occupy the central and positive region of CV1, characterized by reduced anterior canal curvature, but they do separate along CV2 and CV3 (figure 6*b*). TC and GB species occupy a more positive region along CV2, displaying greater dorsoventral curvature of the anterior canal, reduced torsion of the posterior canal, and longer crus-commune. TC and GB species separate along CV3 with the TC ecomorph occupying the positive end of CV3 indicating greater curvature of the lateral canal and reduced curvature of the posterior canal. The remaining ‘trunk’ ecomorphs separate along CV2, with the TG species occupying a more negative region of CV2, representing reduced torsion of the canals in general.

**Table 3.** Posterior probabilities of ecomorph groups being significantly different from one another based on Mahalanobis distances using 10 000 permutations. (CG, crown-giant; GB, grass-bush; TC, trunk-crown; TG, trunk-ground; Tr, trunk; Tw, twig.)

probabilities from Mahalanobis distances						
	CG	GB	TC	TG	Tr	Tw
CG		<0.0001	<0.0001	<0.0001	<0.0001	<0.0001
GB	<0.0001		<0.0001	<0.0001	<0.0001	<0.0001
TC	<0.0001	<0.0001		<0.0001	<0.0001	<0.0001
TG	<0.0001	<0.0001	<0.0001		<0.0001	<0.0001
Tr	<0.0001	<0.0001	<0.0001	<0.0001		<0.0001
Tw	<0.0001	<0.0001	<0.0001	<0.0001	<0.0001	

The unique species (those that have not been assigned to any of the ecomorphs in previous studies) are spread throughout morphospace (figure 6), with only some visually falling within the 95% CI of modern ecomorphs in the first few axes (CV1–3). Posterior probabilities of Mahalanobis distances over all CVs find that many unique species fall significantly outside the 95% CIs defined by the ecomorphs, with exceptions (electronic supplementary material, table S5): (i) one specimen of *A. argenteolus* falls within the 95% CI of TC (*p* = 0.139); (ii) the sole *A. bartschi* specimen falls within the 95% CI of TC (*p* = 0.895); (iii) out of five *A. christophei* specimens, one falls within the 95% CI of TG (*p* = 0.223), one in TC (*p* = 0.151), and one in GB (*p* = 0.073); (iv) out of five *A. etheridgei* specimens, two fall within the 95% CI of TG (*p* = 0.191, *p* = 0.052); (v) out of five *A. monticola* specimens, three fall within the 95% CI of TG (*p* = 0.209, *p* = 0.075, *p* = 0.348) and one in Tr (*p* = 0.103); (vi) the only *A. reconditus* falls within TG (*p* = 0.124); and (vii) out of four *A. rimarum* specimens, one falls within the 95% CI of GB (*p* = 0.478) and one in TC (*p* = 0.059). These results indicate that unique species display greater variation in semicircular canal shape than what is encompassed by the ecomorph categories. Our log-likelihood calculations do, however, assign all unique species to the defined ecomorphs, although these assignments are also inconsistent within and between species (electronic supplementary material, table S5).

All the fossil specimens fall either outside or just on the margin of the 95% CI of the modern ecomorphs, much like the unique species (figure 6). Our posterior probabilities support this: all fossil specimens are highly unlikely to belong to any modern ecomorph group (table 4). This contrasts with the log-likelihood tests which assign each fossil to the ‘closest’ ecomorph group regardless of actual morphological distance (table 4). Moreover, log-likelihood tests found only one instance of correspondence with the ecomorphs inferred by Sherratt *et al.* [34]: OAAAA, which is assigned to TC. Visually, all fossils are broadly distributed around the first three CV axes, with some being in extreme regions of morphospace (figure 6). However, M-525 generally falls close to TG (CV1 vs CV2) and Tr (CV2 vs CV3), even though it statistically falls outside their 95% CIs (table 4).

**Table 4.** Probabilities and log-likelihoods of fossil anoles belonging to modern ecomorph groups, compared with the results of Sherratt *et al*. [34]. (Probabilities below 0.05 indicate the fossil is significantly different from an ecomorph group. Likelihoods are calculated as the most-likely modern group without an alternative hypothesis (i.e. fossils are unique). CG, crown-giant; GB, grass-bush; TC, trunk-crown; TG, trunk-ground; Tr, trunk; Tw, twig.)

		probability						
	log-likelihood	CG	GB	TC	TG	Tr	Tw	Sherratt *et al*. [34] log-likelihood
M-1153	GB (0.99)	<0.0001	0.0313	<0.0001	<0.0001	<0.0001	<0.0001	TC (1.00)
M-3410	GB (0.99)	<0.0001	0.0108	<0.0001	<0.0001	<0.0001	<0.0001	TC (0.69)
M-525	TW (0.90)	<0.0001	<0.0001	0.0002	<0.0001	<0.0001	0.0018	TC (0.99)
OAAAA	TC (0.99)	<0.0001	<0.0001	0.0086	<0.0001	<0.0001	<0.0001	TC (1.00)
USNM580060	CG (0.21)	<0.0001	<0.0001	<0.0001	<0.0001	<0.0001	<0.0001	TG (0.99)

## 4. Discussion

### 4.1. Convergence of semicircular canal shape

Our results support the hypothesis that phylogenetically disparate *Anolis* species have convergent semicircular canal morphologies, allowing them to navigate similar ecological niches. Of the covariates we tested (ecomorph, size, head proportions), we found ecomorph grouping to be the best determinant of canal shape, even when phylogeny is accounted for (25–35%, tables 1 and 2). This is remarkable considering the taxa we examined constitute only a single genus which inhabits a relatively constrained geographical and ecological space. However, given the role semicircular canals play in coordinating fast and complex movements in 3D, and the differences in ecology and locomotor behaviour among the *Anolis* ecomorphs, this result aligns well with the body of work supporting a relationship between semicircular canal morphology and locomotion [5,7,9,22,27,28].

Though differences among the ecomorphs are subtle, CVA posterior probabilities found that all ecomorphs form significantly different groupings, though our ANCOVA *post hoc* pairwise comparisons did not find a statistical difference between TG and Tr ecomorphs (electronic supplementary material, table S3). While CV1 and 2 do not discriminate all groups, all axes of variation must be considered to properly establish group separation [67]. Why our CVA results and *post hoc* tests do not fully align is uncertain—both statistical tests use similar non-parametric methods, though use different distant measures (Procrustes [69] versus Mahalanobis [61]). Of all the groups, Tr and TG ecomorphs are certainly the most similar—demonstrated by their overlap in CVA morphospace (figure 6), which may reflect locomotor/behavioural similarities.

Both CG and Tw ecomorphs display more torsion of the anterior canal than those of the other ecomorphs, separating significantly along CV1 (figure 6*a*): CG and Tw ecomorphs are the two groups that generally run the least, but also have to negotiate extremely complicated 3D environments. In mammals, increasing out-of-plane torsion of a canal may increase sensitivity to rotations out of the canal's major plane of motion detection [64]; thus such a morphology in CG and Tw ecomorphs may potentially reflect the coordination required to negotiate their complex environment. Further, CV3 groups the three most arboreal ecomorphs: TCs alongside CG and Tw based on increased circularity and length of the lateral canal (figure 6). Both increased canal circularity [5] and length [3] have been associated with greater canal sensitivity and agility in mammals. These potential increases in sensitivity of both anterior and lateral canals may represent adaptations to the specialized arboreal niches of these three ecomorphs—CG, Tw and TC—occupying the complex upper reaches of the canopy, requiring greater sensitivity to movements. For the remaining ecomorphs, out of plane sensitivity may not be as essential to locomotor performance. For Tr and TG ecomorphs the trunk provides a broad, uncomplicated surface on which locomotion is much easier [70–72], requiring less refined balance. The perch diameter for GB ecomorphs is indeed relatively much more narrow [33,73] and complex, though the consequences of falling from grass or a bush are far less severe than falling from the tree canopy as in the higher dwelling ecomorphs. Perhaps these relaxed locomotory constraints result in less drastic semicircular canal specialization among Tr, TG and GB ecomorphs.

Generally, however, the ecomorphological signal we found does not fully explain variation in semicircular canal shape. Analysis of canal shape and perch height and diameter returned non-significant results (tables 1 and 2), despite both being correlated with ecomorph [33]. This finding was unexpected given the importance of balance during locomotion on narrow perches, and the assumed consequences of falling from high perches. The perch data included here are from a different population than our morphological data; perhaps this limitation introduced sufficient error into our analysis to confound the relationship. Further work is needed. Other behavioural characteristics may also be associated with canal shape variation, such as locomotor performance over varied substrates and/or head rotational velocities [19], and we encourage collection of such data. The remaining variation in canal morphology may also be the result of morphological ‘noise’ introduced by the skull. As the morphology of the semicircular canals must be accommodated by the skull, there may be trade-offs with the other functional requirements of the skull—such as the brain, the feeding apparatus and other senses of sight and hearing. Reduced penalties for locomotory performance in less arboreal ecomorphs may release the skull to accommodate these other vital functions. Although our results do not indicate canal shape is strongly influenced by head proportions (tables 1 and 2), recent explorations of *Anolis* skull morphology using geometric morphometric techniques have established differences in skull shape between ecomorphs [52,74]. Perhaps the semicircular canals are being influenced by covariation of the skull, but our head proportion ratio was not sensitive enough to capture it. Comparisons between semicircular canal shape and multidimensional skull shape should be an interesting route of future inquiry that might reveal additional influences on vestibular anatomy.

### 4.2. Role of phylogeny and size

We found a small yet significant relationship between phylogenetic relatedness and semicircular canal morphology (figure 5; tables 1 and 2). This weak phylogenetic signal may be the result of repeated convergent evolution for which anoles are famous [33], though similarly weak yet significant phylogenetic signals are consistent across studies dealing with other taxonomic groups [28,35–37]. It is also likely that we are simply dealing with limited divergences as we are working within a single genus—previous studies have generally compared broader taxonomic groups [28,35–37]. Based on these significant phylogenetic signals, some authors have suggested using the inner ear as a source of phylogenetic characters [75,76]. However, the results of our study demonstrate that while semicircular canal morphology is related to phylogenetic history, size and ecology are more important factors (tables 1 and 2) and any phylogenetic analysis based on such characters would be unreliable. Billet *et al*. [77] concluded similarly in their phylogenetic analysis of litopternan petrosal and inner ear characters, finding a potentially confounding allometric signal.

Skull length and width (and canal size) is correlated with semicircular canal morphology in *Anolis* and our results also show that it covaries with ecomorph (electronic supplementary material, table S6). This interaction suggests differences among the allometric shape trajectories of the six ecomorph groups. Previous studies have found that canal size appears to scale with negative allometry, such that smaller animals have relatively larger canals [7,9,37,38]. Some have postulated that smaller animals experience relatively greater angular accelerations of the head than do large animals [7,9,78] and that canal sensitivity is tied to canal radius, suggesting that larger canals are more sensitive to rotation [3]. We found that all three size metrics were highly correlated with canal morphology (tables 1 and 2; electronic supplementary material, table S6), though negative (or positive) allometry cannot be determined when the response variable (canal shape) is multivariate. However, regression of log centroid size on skull length in our dataset found evidence of strong negative allometry (slope = 0.63; 95% CI = 0.54–0.71) which is in keeping with prior studies. Further analyses exploring the allometric variation in our data will be the subject of future publications.

### 4.3. Affinities of fossil anoles

Although further research is needed to determine other factors that may covary with semicircular canal morphology, the significant relationship between ecomorph and canal shape in extant *Anolis* species enabled us to explore the palaeoecology of fossil taxa. Using posterior (typicality) probabilities, we find that the semicircular canal shapes of all five fossils are significantly different from modern ecomorph groupings (table 4), and that all five also differ from each other (figures 2, 4 and 6). It is not unreasonable for the fossil taxa to differ from modern morphological patterns: Anoles probably first reached Hispaniola in the late Eocene approximately 40 Ma [34], so these 20 Ma Miocene fossils probably represent an intermediate period of diversification between Eocene and modern anoles. Further, the ecological context of Miocene anoles was probably different from the modern Antillean ecosystems. Though little is known about the forest ecosystem structure of the Antilles during the Miocene, *Hymenaea protera*, the amber forming tree in which the fossils are contained, is more closely related to the African *Hymenaea verrucosa* than the modern Antillean species [40,79]. Differences in floral composition in the Miocene may have influenced how extinct anoles were navigating their island environment, meaning semicircular canal shape may have been under different selective pressures.

Using a log-likelihood approach (table 4), the fossil anoles are sorted into modern ecomorph groupings, however, only OAAAA matches the predicted groupings of Sherratt *et al.* [34] who used external morphological features to define ecomorphs. It is possible that the discrepancy between the two studies is a result of quantifying different anatomical structures. Taphonomic distortion may also be an unavoidable factor in fossil specimens. Of the five fossil anoles, two fall into an extreme region of PCA morphospace (figure 4), which could imply taphonomic distortion. However, close inspection of the fossils finds that only M-525 has any noticeable deformation of the basicranium (lateral compression, figure 2). Therefore, we do not expect taphonomic distortion to be causing these differences between studies. Alternatively, the discrepancy between our study and Sherratt *et al.* [34] may be owing to mosaic evolution: the vestibular system may responded differently to selective pressures than other ecomorphological traits (e.g. limb length, digit length, subdigital lamellae) resulting in varying rates of morphological change [80].

## 5. Conclusion

Here we demonstrate that the classic ecomorph definitions of *Anolis* of the Greater Antilles are supported by inner ear morphology, with each ecomorph possessing a distinctive semicircular canal shape. We find that, of the covariates we tested, ecomorph is the single-most important covariate of canal morphology, although phylogenetic history, canal size and head proportions are also significantly correlated with canal shape. Surprisingly, we were unable to find any correlation between canal shape and perch variables; this result may suggest that canal shape is not influenced by where anoles live, but rather how they locomote. Still, much of the morphological variance seen in our sample remains unexplained and further work is required to tease out other ecological, behavioural, and/or anatomical characteristics that may covary with semicircular canal morphology. Using the more conservative metric of posterior (typicality) probabilities, we were unable to assign fossil anoles to modern ecomorph groups based upon semicircular canal shape. Our results indicate that the semicircular canals of these extinct anoles are morphologically different from modern *Anolis* ecomorphs, suggesting fossil taxa may have been interacting with their Miocene environment in different ways to modern *Anolis* species.

## Supplementary Material

Sup. 1. Supplementary figures and tables.docx

## Data accessibility

All data and code for analyses are available in the Dryad data repository at the following link: http://dx.doi.org/10.5061/dryad.8s586 [81].
