# Supplementary material for: Semicircular canals in Anolis lizards: ecomorphological convergence and ecomorph affinities of fossil species
Source: R Soc Open Sci. 2017 Oct 11;4(10):170058. doi: 10.1098/rsos.170058 (PMC5666239; doi:10.1098/rsos.170058)
Supplement: Sup. 1. Supplementary figures and tables.docx [file rsos170058supp1.docx]

**Supplementary material**

**Table S1.** List of specimens with collection ID, µCT scan details, ecomorph, island of origin and skull dimensions. Anole specimens are from the *Anolis* and *Chamaelinorops* genera. Collection IDs refer to museum accession numbers. All modern anole species are housed in the Herpetology Department, Museum of Comparative Zoology, Harvard. µCT scans performed at the following institutes: MCZ: Museum of Comparative Zoology, Harvard (Skyscan 1173); CNS: Harvard Centre for Nanoscale Systems (Nikon Metrology [X-Tek] HMXST225); UT: University of Texas High-Resolution X-ray CT Facility; IAC-NHM: Imaging and Analysis Centre, Natural History Museum London; USNM: Smithsonian Institution National Museum of Natural History, Washington DC.

| Species | Collection ID | µCT scan | ecomorph | Island | skull length (mm) | skull width (mm) |
| --- | --- | --- | --- | --- | --- | --- |
| *A. allisoni* | MCZ:Herp:R60917 | MCZ | trunk-crown | Cuba | 16.52 | 5.86 |
| *A. allisoni* | MCZ:Herp:R60919 | MCZ | trunk-crown | Cuba | 15.34 | 5.74 |
| *A. alutaceus* | MCZ:Herp:R52457 | MCZ | grass-bush | Cuba | 10.54 | 3.77 |
| *A. angusticeps* | MCZ:Herp:R59254 | MCZ | twig | Cuba | 14.84 | 5.47 |
| *A. argenteolus* | MCZ:Herp:R93474 | MCZ | unique | Cuba | 15.01 | 5.37 |
| *A. argillaceus* | MCZ:Herp:R17122 | MCZ | unique | Cuba | 12.14 | 5.10 |
| *A. bahorucoensis* | MCZ:Herp:R143546 | CNS | grass-bush | Hispaniola | 12.28 | 4.49 |
| *A. bahorucoensis* | MCZ:Herp:R186720 | CNS | grass-bush | Hispaniola | 11.40 | 4.30 |
| *A. bahorucoensis* | MCZ:Herp:R187465 | CNS | grass-bush | Hispaniola | 14.66 | 5.20 |
| *A. bahorucoensis* | MCZ:Herp:R187482 | CNS | grass-bush | Hispaniola | 9.40 | 3.72 |
| *A. baleatus* | MCZ:Herp:R132301 | CNS | crown-giant | Hispaniola | 18.13 | 6.96 |
| *A. baleatus* | MCZ:Herp:R186658 | CNS | crown-giant | Hispaniola | 20.36 | 7.78 |
| *A. baleatus* | MCZ:Herp:R186660 | CNS | crown-giant | Hispaniola | 22.38 | 8.22 |
| *A. baleatus* | MCZ:Herp:R186661 | CNS | crown-giant | Hispaniola | 20.04 | 7.84 |
| *A. baleatus* | MCZ:Herp:R186662 | CNS | crown-giant | Hispaniola | 18.14 | 6.94 |
| *A. baleatus* | MCZ:Herp:R186663 | CNS | crown-giant | Hispaniola | 15.03 | 6.36 |
| *A. barahonae* | MCZ:Herp:R171757 | CNS | crown-giant | Hispaniola | 18.04 | 7.41 |
| *A. barahonae* | MCZ:Herp:R182401 | CNS | crown-giant | Hispaniola | 19.93 | 7.59 |
| *A. bartschi* | MCZ:Herp:R38420 | MCZ | unique | Cuba | 19.79 | 7.53 |
| *A. brevirostris* | MCZ:Herp:R68689 | CNS | trunk | Hispaniola | 12.92 | 6.05 |
| *A. brevirostris* | MCZ:Herp:R155829 | CNS | trunk | Hispaniola | 14.07 | 6.71 |
| *A. brevirostris* | MCZ:Herp:R155833 | CNS | trunk | Hispaniola | 9.59 | 4.67 |
| *A. brevirostris* | MCZ:Herp:R155836 | CNS | trunk | Hispaniola | 7.69 | 3.99 |
| *A. brevirostris* | MCZ:Herp:R155845 | CNS | trunk | Hispaniola | 7.27 | 3.79 |
| *A. brevirostris* | MCZ:Herp:R69274 | CNS | trunk | Hispaniola | 12.34 | 5.97 |
| *A. brunneus* | MCZ:Herp:R183258 | MCZ | trunk-crown | Bahamas | 14.43 | 5.30 |
| *A. chlorocyanus* | MCZ:Herp:R158041 | CNS | trunk-crown | Hispaniola | 15.27 | 6.24 |
| *A. chlorocyanus* | MCZ:Herp:R159397 | CNS | trunk-crown | Hispaniola | 11.14 | 4.61 |
| *A. chlorocyanus* | MCZ:Herp:R159654 | CNS | trunk-crown | Hispaniola | 12.15 | 4.89 |
| *A. chlorocyanus* | MCZ:Herp:R186903 | CNS | trunk-crown | Hispaniola | 17.06 | 6.95 |
| *A. chlorocyanus* | MCZ:Herp:R186950 | CNS | trunk-crown | Hispaniola | 16.16 | 6.56 |
| *A. chlorocyanus* | MCZ:Herp:R187268 | CNS | trunk-crown | Hispaniola | 14.20 | 5.65 |
| *A. chlorocyanus* | MCZ:Herp:R59788 | CNS | trunk-crown | Hispaniola | 12.44 | 4.82 |
| *A. chlorocyanus* | MCZ:Herp:R62922 | CNS | trunk-crown | Hispaniola | 9.14 | 3.63 |
| *A. christophei* | MCZ:Herp:R107066 | CNS | unique | Hispaniola | 11.63 | 4.75 |
| *A. christophei* | MCZ:Herp:R186671 | CNS | unique | Hispaniola | 9.13 | 3.95 |
| *A. christophei* | MCZ:Herp:R186674 | CNS | unique | Hispaniola | 12.39 | 5.10 |
| *A. christophei* | MCZ:Herp:R186693 | CNS | unique | Hispaniola | 14.13 | 5.60 |
| *A. christophei* | MCZ:Herp:R79360 | CNS | unique | Hispaniola | 10.39 | 4.30 |
| *A. coelestinus* | MCZ:Herp:R187468 | CNS | trunk-crown | Hispaniola | 14.35 | 5.49 |
| *A. coelestinus* | MCZ:Herp:R57527 | CNS | trunk-crown | Hispaniola | 14.97 | 5.89 |
| *A. coelestinus* | MCZ:Herp:R64542 | CNS | trunk-crown | Hispaniola | 14.12 | 5.41 |
| *A. coelestinus* | MCZ:Herp:R64547 | CNS | trunk-crown | Hispaniola | 11.61 | 4.37 |
| *A. cristatellus* | MCZ:Herp:R96257 | MCZ | trunk-ground | Puerto Rico | 17.33 | 7.91 |
| *A. cuvieri* | MCZ:Herp:R57898 | MCZ | crown-giant | Puerto Rico | 37.00 | 15.46 |
| *A. cybotes* | MCZ:Herp:R171762 | CNS | trunk-ground | Hispaniola | 14.09 | 6.87 |
| *A. cybotes* | MCZ:Herp:R186752 | CNS | trunk-ground | Hispaniola | 13.18 | 5.90 |
| *A. cybotes* | MCZ:Herp:R187014 | CNS | trunk-ground | Hispaniola | 11.42 | 5.38 |
| *A. cybotes* | MCZ:Herp:R57569 | CNS | trunk-ground | Hispaniola | 11.70 | 5.36 |
| *A. cybotes* | MCZ:Herp:R57575 | CNS | trunk-ground | Hispaniola | 7.54 | 4.09 |
| *A. cybotes* | MCZ:Herp:R57624 | CNS | trunk-ground | Hispaniola | 13.74 | 6.70 |
| *A. cybotes* | MCZ:Herp:R86760 | CNS | trunk-ground | Hispaniola | 15.93 | 7.30 |
| *A. distichus* | MCZ:Herp:R158151 | CNS | trunk | Hispaniola | 8.44 | 4.19 |
| *A. distichus* | MCZ:Herp:R187075 | CNS | trunk | Hispaniola | 13.31 | 6.39 |
| *A. distichus* | MCZ:Herp:R187141 | CNS | trunk | Hispaniola | 13.05 | 6.13 |
| *A. distichus* | MCZ:Herp:R187194 | CNS | trunk | Hispaniola | 10.81 | 5.31 |
| *A. distichus* | MCZ:Herp:R187204 | CNS | trunk | Hispaniola | 10.80 | 5.03 |
| *A. distichus* | MCZ:Herp:R58492 | CNS | trunk | Hispaniola | 8.21 | 4.09 |
| *A. dolichocephalus* | MCZ:Herp:R132763 | CNS | grass-bush | Hispaniola | 7.21 | 3.01 |
| *A. dolichocephalus* | MCZ:Herp:R74865 | CNS | grass-bush | Hispaniola | 7.82 | 3.11 |
| *A. equestris* | MCZ:Herp:R55629 | MCZ | crown-giant | Cuba | 42.55 | 16.75 |
| *A. etheridgei* | MCZ:Herp:R107022 | CNS | unique | Hispaniola | 9.49 | 4.11 |
| *A. etheridgei* | MCZ:Herp:R186769 | CNS | unique | Hispaniola | 10.45 | 4.46 |
| *A. etheridgei* | MCZ:Herp:R186770 | CNS | unique | Hispaniola | 11.84 | 5.11 |
| *A. etheridgei* | MCZ:Herp:R186772 | CNS | unique | Hispaniola | 12.63 | 5.37 |
| *A. etheridgei* | MCZ:Herp:R186781 | CNS | unique | Hispaniola | 10.66 | 4.43 |
| *A. etheridgei* | MCZ:Herp:R79347 | CNS | unique | Hispaniola | 8.45 | 3.88 |
| *A. evermanni* | MCZ:Herp:R61811 | MCZ | trunk-crown | Puerto Rico | 17.63 | 7.62 |
| *A. garmani* | MCZ:Herp:R158578 | MCZ | crown-giant | Jamaica | 28.37 | 11.85 |
| *A. grahami* | MCZ:Herp:R75433 | MCZ | trunk-crown | Jamaica | 18.52 | 7.79 |
| *A. hendersoni* | MCZ:Herp:R62963 | CNS | grass-bush | Hispaniola | 11.84 | 4.23 |
| *A. hendersoni* | MCZ:Herp:R65639 | CNS | grass-bush | Hispaniola | 11.95 | 4.31 |
| *A. hendersoni* | MCZ:Herp:R65643 | CNS | grass-bush | Hispaniola | 11.61 | 4.10 |
| *A. insolitus* | MCZ:Herp:R107014 | CNS | twig | Hispaniola | 11.26 | 3.53 |
| *A. insolitus* | MCZ:Herp:R107016 | CNS | twig | Hispaniola | 6.37 | 2.50 |
| *A. insolitus* | MCZ:Herp:R128310 | CNS | twig | Hispaniola | 11.70 | 3.63 |
| *A. insolitus* | MCZ:Herp:R186694 | CNS | twig | Hispaniola | 11.68 | 3.71 |
| *A. insolitus* | MCZ:Herp:R187100 | CNS | twig | Hispaniola | 13.69 | 4.24 |
| *A. lineatopis* | MCZ:Herp:R192710 | MCZ | trunk-ground | Puerto Rico | 16.90 | 6.62 |
| *A. longiceps* | MCZ:Herp:R16190 | CNS | trunk-crown | Hispaniola | 15.99 | 5.73 |
| *A. longiceps* | MCZ:Herp:R16194 | CNS | trunk-crown | Hispaniola | 14.67 | 5.28 |
| *A. longiceps* | MCZ:Herp:R16830 | CNS | trunk-crown | Hispaniola | 9.36 | 3.95 |
| *A. longiceps* | MCZ:Herp:R189078 | CNS | trunk-crown | Hispaniola | 9.63 | 3.96 |
| *A. loysiana* | MCZ:Herp:R74042 | MCZ | trunk | Cuba | 10.81 | 4.78 |
| *A. marcanoi* | MCZ:Herp:R104402 | CNS | trunk-ground | Hispaniola | 12.22 | 5.51 |
| *A. marcanoi* | MCZ:Herp:R104403 | CNS | trunk-ground | Hispaniola | 12.28 | 5.58 |
| *A. marcanoi* | MCZ:Herp:R131841 | CNS | trunk-ground | Hispaniola | 7.74 | 3.98 |
| *A. marcanoi* | MCZ:Herp:R143255 | CNS | trunk-ground | Hispaniola | 13.68 | 6.02 |
| *A. marcanoi* | MCZ:Herp:R143442 | CNS | trunk-ground | Hispaniola | 10.45 | 4.76 |
| *A. marcanoi* | MCZ:Herp:R143443 | CNS | trunk-ground | Hispaniola | 9.02 | 4.08 |
| *A. marcanoi* | MCZ:Herp:R150528 | CNS | trunk-ground | Hispaniola | 13.95 | 5.98 |
| *A. monticola* | MCZ:Herp:R124871 | CNS | unique | Hispaniola | 11.01 | 4.45 |
| *A. monticola* | MCZ:Herp:R124872 | CNS | unique | Hispaniola | 10.48 | 4.49 |
| *A. monticola* | MCZ:Herp:R124876 | CNS | unique | Hispaniola | 12.57 | 5.13 |
| *A. monticola* | MCZ:Herp:R124900 | CNS | unique | Hispaniola | 12.00 | 4.92 |
| *A. monticola* | MCZ:Herp:R127720 | CNS | unique | Hispaniola | 8.22 | 3.67 |
| *A. occultus* | MCZ:Herp:R101804 | CNS | twig | Puerto Rico | 11.27 | 3.64 |
| *A. occultus* | MCZ:Herp:R101808 | CNS | twig | Puerto Rico | 10.90 | 3.50 |
| *A. occultus* | MCZ:Herp:R83658 | CNS | twig | Puerto Rico | 6.37 | 2.45 |
| *A. occultus* | MCZ:Herp:R83662 | CNS | twig | Puerto Rico | 10.78 | 3.53 |
| *A. olssoni* | MCZ:Herp:R131135 | CNS | grass-bush | Hispaniola | 9.56 | 3.63 |
| *A. olssoni* | MCZ:Herp:R143274 | CNS | grass-bush | Hispaniola | 8.70 | 3.29 |
| *A. olssoni* | MCZ:Herp:R187437 | CNS | grass-bush | Hispaniola | 10.88 | 4.06 |
| *A. olssoni* | MCZ:Herp:R187439 | CNS | grass-bush | Hispaniola | 12.50 | 4.86 |
| *A. olssoni* | MCZ:Herp:R187483 | CNS | grass-bush | Hispaniola | 11.79 | 4.23 |
| *A. olssoni* | MCZ:Herp:R79313 | CNS | grass-bush | Hispaniola | 10.11 | 3.65 |
| *A. porcatus* | MCZ:Herp:R141418 | MCZ | trunk-crown | Cuba | 17.05 | 6.24 |
| *A. porcatus* | MCZ:Herp:R67388 | MCZ | trunk-crown | Cuba | 17.02 | 6.19 |
| *A. pulchellus* | MCZ:Herp:R100133 | MCZ | grass-bush | Puerto Rico | 14.17 | 5.07 |
| *A. reconditus* | MCZ:Herp:R122270 | MCZ | unique | Jamaica | 19.44 | 8.01 |
| *A. ricordii* | MCZ:Herp:R83982 | CNS | crown-giant | Hispaniola | 16.95 | 6.55 |
| *A. rimarum* | MCZ:Herp:R124657 | CNS | unique | Hispaniola | 9.43 | 4.05 |
| *A. rimarum* | MCZ:Herp:R124660 | CNS | unique | Hispaniola | 10.99 | 4.54 |
| *A. rimarum* | MCZ:Herp:R81128 | CNS | unique | Hispaniola | 12.69 | 5.23 |
| *A. rimarum* | MCZ:Herp:R81129 | CNS | unique | Hispaniola | 10.98 | 4.45 |
| *A. sheplani* | MCZ:Herp:R125641 | CNS | twig | Hispaniola | 12.17 | 3.37 |
| *A. sheplani* | MCZ:Herp:R125642 | CNS | twig | Hispaniola | 10.89 | 3.23 |
| *A. singularis* | MCZ:Herp:R68656 | CNS | trunk-crown | Hispaniola | 14.76 | 5.62 |
| *A. singularis* | MCZ:Herp:R143383 | CNS | trunk-crown | Hispaniola | 13.04 | 5.47 |
| *A. singularis* | MCZ:Herp:R150005 | CNS | trunk-crown | Hispaniola | 14.59 | 5.93 |
| *A. singularis* | MCZ:Herp:R167626 | CNS | trunk-crown | Hispaniola | 11.01 | 4.40 |
| *A. singularis* | MCZ:Herp:R186713 | CNS | trunk-crown | Hispaniola | 11.06 | 4.36 |
| *A. valencienni* | MCZ:Herp:R93449 | MCZ | twig | Jamaica | 21.18 | 8.08 |
| *C. barbouri* | MCZ:Herp:R170143 | CNS | unique | Hispaniola | 6.63 | 3.35 |
| *C. barbouri* | MCZ:Herp:R170148 | CNS | unique | Hispaniola | 6.65 | 3.35 |
| *C. barbouri* | MCZ:Herp:R170156 | CNS | unique | Hispaniola | 8.15 | 3.76 |
| *C. barbouri* | MCZ:Herp:R171029 | CNS | unique | Hispaniola | 10.10 | 4.17 |
| *C. barbouri* | MCZ:Herp:R171031 | CNS | unique | Hispaniola | 10.78 | 4.46 |
| *C. barbouri* | MCZ:Herp:R171036 | CNS | unique | Hispaniola | 8.61 | 3.78 |
| *C. barbouri* | MCZ:Herp:R171038 | CNS | unique | Hispaniola | 11.07 | 4.46 |
| *C. barbouri* | MCZ:Herp:R171040 | CNS | unique | Hispaniola | 10.19 | 4.18 |
| M-1153 | Private Collection (Sherratt et al. 2015) | IAC-NHM | Fossil | Hispaniola | 8.40 | 4.88 |
| M-3410 | Private Collection (Sherratt et al. 2015) | IAC-NHM | Fossil | Hispaniola | 7.40 | - |
| M-525 | Private Collection (Sherratt et al. 2015) | IAC-NHM | Fossil | Hispaniola | 6.43 | 3.80 |
| OAAAA | Private Collection (Sherratt et al. 2015) | CNS | Fossil | Hispaniola | 6.57 | 3.91 |
| USNM580060 | USNM580060 | UT | Fossil | Hispaniola | 6.57 | 3.63 |

**Table S2.** MANOVA of ecomorph vs shape across all Principal Components. Ecomorphs are significantly different over PCs 2-8, which represent 42.5% of shape variation.

|  | % Var | p |  | % Var | p |  | % Var | p |
| --- | --- | --- | --- | --- | --- | --- | --- | --- |
| PC1 | 0.432 | 0.234 | PC30 | 0.002 | 0.853 | PC60 | <0.001 | 1.000 |
| **PC2** | **0.128** | **<0.001** | PC31 | 0.001 | 0.776 | PC61 | <0.001 | 0.858 |
| **PC3** | **0.089** | **<0.001** | PC32 | 0.001 | 0.380 | PC62 | <0.001 | 0.999 |
| **PC4** | **0.067** | **<0.001** | PC33 | 0.001 | 0.278 | PC63 | <0.001 | 0.761 |
| **PC5** | **0.049** | **0.007** | PC34 | 0.001 | 0.526 | PC64 | <0.001 | 0.984 |
| **PC6** | **0.038** | **0.012** | PC35 | 0.001 | 0.971 | PC65 | <0.001 | 0.979 |
| **PC7** | **0.030** | **0.001** | PC36 | 0.001 | 0.942 | PC66 | <0.001 | 0.864 |
| **PC8** | **0.022** | **<0.001** | PC37 | 0.001 | 0.568 | PC67 | <0.001 | 0.873 |
| PC9 | 0.016 | 0.828 | PC38 | 0.001 | 0.526 | PC68 | <0.001 | 0.927 |
| PC10 | 0.015 | 0.843 | PC39 | 0.001 | 0.931 | PC69 | <0.001 | 0.888 |
| PC11 | 0.011 | 0.409 | PC40 | 0.001 | 0.987 | PC70 | <0.001 | 0.689 |
| PC12 | 0.009 | 0.427 | PC41 | 0.001 | 0.800 | PC71 | <0.001 | 0.811 |
| PC13 | 0.009 | 0.950 | PC42 | 0.001 | 0.938 | PC72 | <0.001 | 0.940 |
| PC14 | 0.007 | 0.293 | PC43 | 0.001 | 0.856 | PC73 | <0.001 | 1.000 |
| PC15 | 0.007 | 0.982 | PC44 | <0.001 | 0.955 | PC74 | <0.001 | 0.895 |
| PC16 | 0.006 | 0.274 | PC45 | <0.001 | 0.925 | PC75 | <0.001 | 0.913 |
| PC17 | 0.006 | 0.136 | PC46 | <0.001 | 0.879 | PC76 | <0.001 | 0.904 |
| PC18 | 0.005 | 0.335 | PC47 | <0.001 | 0.609 | PC77 | <0.001 | 0.561 |
| PC19 | 0.005 | 0.088 | PC48 | <0.001 | 0.704 | PC78 | <0.001 | 0.676 |
| PC20 | 0.004 | 0.996 | PC49 | <0.001 | 0.992 | PC79 | <0.001 | 0.654 |
| PC21 | 0.004 | 0.796 | PC50 | <0.001 | 0.995 | PC80 | <0.001 | 0.840 |
| PC22 | 0.004 | 0.286 | PC51 | <0.001 | 0.946 | PC81 | <0.001 | 0.849 |
| PC23 | 0.003 | 0.801 | PC52 | <0.001 | 0.936 | PC82 | <0.001 | 0.999 |
| PC24 | 0.003 | 0.805 | PC53 | <0.001 | 0.797 | PC83 | <0.001 | 0.898 |
| PC25 | 0.002 | 0.456 | PC54 | <0.001 | 0.994 | PC84 | <0.001 | 0.986 |
| PC26 | 0.002 | 0.217 | PC55 | <0.001 | 0.735 | PC85 | <0.001 | 0.956 |
| PC27 | 0.002 | 0.056 | PC56 | <0.001 | 0.935 | PC86 | <0.001 | 0.983 |
| PC28 | 0.002 | 0.673 | PC57 | <0.001 | 0.620 | PC87 | <0.001 | 0.995 |
| PC29 | 0.002 | 0.238 | PC58 | <0.001 | 0.985 |  |  |  |
| PC30 | 0.002 | 0.853 | PC59 | <0.001 | 0.893 |  |  |  |

**Table S3.** Post-hoc test for pairwise significance between ecomorph groups. CG=crown-giant, GB=grass-bush, TC=trunk-crown, TG=trunk-ground, Tr=trunk, Tw=twig.

| Post-hoc pairwise p-values | | | | | | |
| --- | --- | --- | --- | --- | --- | --- |
|  | CG | GB | Tr | TC | TG | Tw |
| CG |  | **<0.001** | **<0.001** | **0.001** | **0.002** | **<0.001** |
| GB | **<0.001** |  | **0.034** | **0.024** | **0.004** | **0.001** |
| Tr | **<0.001** | **0.034** |  | **0.018** | 0.300 | **<0.001** |
| TC | **<0.001** | **0.024** | **<0.001** |  | **0.004** | **<0.001** |
| TG | **0.002** | **0.004** | 0.300 | **0.004** |  | **<0.001** |
| Tw | **<0.001** | **0.001** | **<0.001** | **<0.001** | **<0.001** |  |

**Table S4.** CVA Jackknife Cross-validation of ecomorph groups showing pairwise classification accuracy under randomized group resampling. CG=crown-giant, GB=grass-bush, TC=trunk-crown, TG=trunk-ground, Tr=trunk, Tw=twig.

| cross-validated classification result in % | | | | | | |
| --- | --- | --- | --- | --- | --- | --- |
|  | CG | GB | TC | TG | Tr | Tw |
| CG | **91.6667** | 0 | 8.3333 | 0 | 0 | 0 |
| GB | 0 | **88.2353** | 5.8824 | 0 | 0 | 5.8824 |
| TC | 0 | 3.5714 | **89.2857** | 0 | 7.1429 | 0 |
| TG | 0 | 6.25 | 6.25 | **87.5** | 0 | 0 |
| Tr | 0 | 7.6923 | 0 | 7.6923 | **84.6154** | 0 |
| Tw | 7.6923 | 0 | 7.6923 | 0 | 0 | **84.6154** |
| **Overall classification accuracy: 87.87879 %** | | | | | | |

**Table S5.** Probabilities and log-Likelihoods (bold) of unique taxa belonging to an ecomorph, calculated using Mahalanobis distances with statistical significance assessed through 10,000 permutations. CG=crown-giant, GB=grass-bush, TC=trunk-crown, TG=trunk-ground, Tr=trunk,Tw=twig. Csize = semicircular canal centroid size.

| Species | ID | Csize | CG | | GB | | TC | | TG | | Tr | | Tw | |
| --- | --- | --- | --- | --- | --- | --- | --- | --- | --- | --- | --- | --- | --- | --- |
| *A. argenteolus* | R93474 | 9.57 | <0.0001 | **0.0001** | <0.0001 | **0.0001** | 0.1385 | **0.9994** | <0.0001 | **0.0001** | <0.0001 | **0.0001** | <0.0001 | **0.0001** |
| *A. argillaceus* | R17122 | 8.77 | <0.0001 | **0.0006** | <0.0001 | **0.0004** | 0.0101 | **0.3741** | <0.0001 | **0.0005** | 0.0169 | **0.6238** | <0.0001 | **0.0006** |
| *A. bartschi* | R38420 | 12.65 | <0.0001 | **<0.0001** | <0.0001 | **<0.0001** | 0.8950 | **0.9999** | <0.0001 | **<0.0001** | <0.0001 | **<0.0001** | <0.0001 | **<0.0001** |
| *A. christophei* | R107066 | 8.49 | <0.0001 | **0.0007** | <0.0001 | **0.0005** | 0.0242 | **0.9407** | 0.0015 | **0.0570** | <0.0001 | **0.0006** | <0.0001 | **0.0006** |
| *A. christophei* | R186671 | 7.32 | <0.0001 | **0.0001** | <0.0001 | **0.0001** | 0.0016 | **0.0080** | 0.2224 | **0.9917** | <0.0001 | **0.0001** | <0.0001 | **0.0001** |
| *A. christophei* | R186674 | 9.25 | <0.0001 | **0.0048** | <0.0001 | **0.0034** | 0.0034 | **0.9793** | <0.0001 | **0.0036** | <0.0001 | **0.0044** | <0.0001 | **0.0044** |
| *A. christophei* | R186693 | 10.16 | <0.0001 | **0.0001** | 0.0001 | **0.0005** | 0.1511 | **0.9838** | 0.0022 | **0.0154** | <0.0001 | **0.0001** | <0.0001 | **0.0001** |
| *A. christophei* | R79360 | 8.78 | <0.0001 | **0.0002** | 0.0728 | **0.9731** | <0.0001 | **0.0001** | 0.0019 | **0.0261** | <0.0001 | **0.0002** | <0.0001 | **0.0002** |
| *A. etheridgei* | R107022 | 8.13 | <0.0001 | **0.0001** | <0.0001 | **0.0001** | <0.0001 | **<0.0001** | 0.1911 | **0.9996** | <0.0001 | **0.0001** | <0.0001 | **0.0001** |
| *A. etheridgei* | R186769 | 9.00 | <0.0001 | **0.2114** | <0.0001 | **0.1492** | <0.0001 | **0.0906** | <0.0001 | **0.1585** | <0.0001 | **0.1951** | <0.0001 | **0.1951** |
| *A. etheridgei* | R186770 | 10.07 | <0.0001 | **0.0626** | <0.0001 | **0.0442** | <0.0001 | **0.0268** | 0.0002 | **0.7509** | <0.0001 | **0.0578** | <0.0001 | **0.0578** |
| *A. etheridgei* | R186772 | 10.59 | <0.0001 | **0.0003** | <0.0001 | **0.0002** | 0.0014 | **0.0268** | 0.0521 | **0.9721** | <0.0001 | **0.0003** | <0.0001 | **0.0003** |
| *A. etheridgei* | R186781 | 9.15 | <0.0001 | **0.1179** | <0.0001 | **0.0832** | <0.0001 | **0.0505** | 0.0001 | **0.5306** | <0.0001 | **0.1088** | <0.0001 | **0.1088** |
| *A. etheridgei* | R79347 | 7.60 | <0.0001 | **0.0008** | <0.0001 | **0.0006** | <0.0001 | **0.0003** | 0.0209 | **0.9968** | <0.0001 | **0.0007** | <0.0001 | **0.0007** |
| *A. monticola* | R124871 | 8.69 | <0.0001 | **0.0002** | <0.0001 | **0.0001** | <0.0001 | **0.0001** | <0.0001 | **0.0001** | 0.1031 | **0.9994** | <0.0001 | **0.0002** |
| *A. monticola* | R124872 | 8.45 | <0.0001 | **0.0001** | <0.0001 | **0.0001** | <0.0001 | **<0.0001** | 0.2094 | **0.9997** | <0.0001 | **0.0001** | <0.0001 | **0.0001** |
| *A. monticola* | R124876 | 9.49 | <0.0001 | **0.0002** | <0.0001 | **0.0001** | 0.0087 | **0.1060** | 0.0753 | **0.8933** | <0.0001 | **0.0002** | <0.0001 | **0.0002** |
| *A. monticola* | R124900 | 9.47 | <0.0001 | **0.0001** | <0.0001 | **<0.0001** | 0.0001 | **0.0003** | 0.3480 | **0.9995** | <0.0001 | **0.0001** | <0.0001 | **0.0001** |
| *A. monticola* | R127720 | 7.29 | <0.0001 | **0.0005** | 0.0014 | **0.0435** | 0.0307 | **0.9251** | 0.0008 | **0.0252** | 0.0002 | **0.0052** | <0.0001 | **0.0005** |
| *A. reconditus* | R122270 | 13.72 | <0.0001 | **0.0001** | <0.0001 | **0.0001** | 0.0005 | **0.0040** | 0.1244 | **0.9955** | <0.0001 | **0.0001** | <0.0001 | **0.0001** |
| *A. rimarum* | R124657 | 7.98 | <0.0001 | **<0.0001** | 0.4783 | **0.9890** | 0.0043 | **0.0108** | <0.0001 | **<0.0001** | <0.0001 | **<0.0001** | <0.0001 | **<0.0001** |
| *A. rimarum* | R124660 | 8.73 | <0.0001 | **0.0004** | 0.0004 | **0.0082** | 0.0436 | **0.9544** | <0.0001 | **0.0003** | <0.0001 | **0.0003** | 0.0016 | **0.0365** |
| *A. rimarum* | R81128 | 9.81 | <0.0001 | **0.0004** | <0.0001 | **0.0003** | 0.0369 | **0.7744** | 0.0090 | **0.1916** | <0.0001 | **0.0003** | 0.0016 | **0.0331** |
| *A. rimarum* | R81129 | 8.68 | <0.0001 | **0.0003** | 0.0074 | **0.1134** | 0.0588 | **0.8833** | <0.0001 | **0.0002** | <0.0001 | **0.0002** | 0.0002 | **0.0026** |
| *C. barbouri* | R170143 | 6.58 | <0.0001 | **0.2114** | <0.0001 | **0.1492** | <0.0001 | **0.0906** | <0.0001 | **0.1585** | <0.0001 | **0.1951** | <0.0001 | **0.1951** |
| *C. barbouri* | R170148 | 6.19 | <0.0001 | **0.2114** | <0.0001 | **0.1492** | <0.0001 | **0.0906** | <0.0001 | **0.1585** | <0.0001 | **0.1951** | <0.0001 | **0.1951** |
| *C. barbouri* | R170156 | 7.12 | <0.0001 | **0.2114** | <0.0001 | **0.1492** | <0.0001 | **0.0906** | <0.0001 | **0.1585** | <0.0001 | **0.1951** | <0.0001 | **0.1951** |
| *C. barbouri* | R171029 | 8.28 | <0.0001 | **0.0070** | <0.0001 | **0.0049** | <0.0001 | **0.0030** | <0.0001 | **0.0052** | <0.0001 | **0.0065** | 0.0023 | **0.9734** |
| *C. barbouri* | R171031 | 8.63 | <0.0001 | **0.2114** | <0.0001 | **0.1492** | <0.0001 | **0.0906** | <0.0001 | **0.1585** | <0.0001 | **0.1951** | <0.0001 | **0.1951** |
| *C. barbouri* | R171036 | 7.24 | <0.0001 | **0.2114** | <0.0001 | **0.1492** | <0.0001 | **0.0906** | <0.0001 | **0.1585** | <0.0001 | **0.1951** | <0.0001 | **0.1951** |
| *C. barbouri* | R171038 | 8.74 | <0.0001 | **0.2114** | <0.0001 | **0.1492** | <0.0001 | **0.0906** | <0.0001 | **0.1585** | <0.0001 | **0.1951** | <0.0001 | **0.1951** |
| *C. barbouri* | R171040 | 8.44 | <0.0001 | **0.0013** | <0.0001 | **0.0009** | <0.0001 | **0.0006** | 0.0128 | **0.9948** | <0.0001 | **0.0012** | <0.0001 | **0.0012** |

**Table S6.** Analysis of covariance (ANCOVA) and phylogenetic generalised least squares (PGLS) of semicircular canal shape against ecomorph and skull length and width with statistical significance assessed through 10,000 permutations.

| ANCOVA (ecomorph and skull length) | | | | | | | |
| --- | --- | --- | --- | --- | --- | --- | --- |
|  | df | SS | MS | R^2^ | F | Z | P.value |
| **ecomorph** | **6** | **0.049** | **0.008** | **0.349** | **3.974** | **2.268** | **0.001** |
| **length** | **1** | **0.014** | **0.014** | **0.096** | **6.568** | **3.220** | **0.004** |
| ecomorph:length | 6 | 0.022 | 0.004 | 0.159 | 1.805 | 1.028 | 0.354 |
| Residuals | 27 | 0.056 | 0.002 |  |  |  |  |
| Total | 40 | 0.141 |  |  |  |  |  |
| PGLS (ecomorph and skull length) | | | | | | | |
|  | df | SS | MS | R^2^ | F | Z | P.value |
| **ecomorph** | **6** | **0.168** | **0.028** | **0.246** | **2.602** | **3.425** | **0.001** |
| **length** | **1** | **0.087** | **0.087** | **0.127** | **8.063** | **4.818** | **0.001** |
| **ecomorph:length** | **6** | **0.139** | **0.023** | **0.203** | **2.151** | **1.979** | **0.009** |
| Residuals | 27 | 0.290 | 0.011 |  |  |  |  |
| Total | 40 | 0.684 |  |  |  |  |  |
| ANCOVA (ecomorph and skull width) | | | | | | | |
|  | df | SS | MS | R^2^ | F | Z | P.value |
| **ecomorph** | **6** | **0.049** | **0.008** | **0.349** | **3.789** | **2.289** | **0.001** |
| **width** | **1** | **0.014** | **0.014** | **0.102** | **6.665** | **3.560** | **0.003** |
| ecomorph:width | 6 | 0.019 | 0.003 | 0.133 | 1.445 | 0.865 | 0.662 |
| Residuals | 27 | 0.058 | 0.002 |  |  |  |  |
| Total | 40 |  |  |  |  |  |  |
| PGLS (ecomorph and skull width) | | | | | | | |
|  | df | SS | MS | R^2^ | F | Z | P.value |
| **ecomorph** | **6** | **0.168** | **0.028** | **0.246** | **2.442** | **3.255** | **0.001** |
| **width** | **1** | **0.072** | **0.072** | **0.106** | **6.324** | **5.452** | **0.001** |
| **ecomorph:width** | **6** | **0.134** | **0.022** | **0.196** | **1.949** | **1.995** | **0.007** |
| Residuals | 27 | 0.310 | 0.011 |  |  |  |  |
| Total | 40 | 0.684 |  |  |  |  |  |
